# Artificial Intelligence Applications to Measure Food and Nutrient Intakes: Scoping Review

**DOI:** 10.2196/54557

**Published:** 2024-11-28

**Authors:** Jiakun Zheng, Junjie Wang, Jing Shen, Ruopeng An

**Affiliations:** 1 School of Economics and Management Shanghai University of Sport Shanghai China; 2 School of Kinesiology and Health Promotion Dalian University of Technology Dalian China; 3 Department of Physical Education China University of Geosciences (Beijing) Beijing China; 4 Silver School of Social Work New York University New York, NY United States

**Keywords:** food, nutrient, diet, artificial intelligence, machine learning, deep learning, neural networks, computer vision, natural language processing, measurement, AI, food intake, systematic literature, dietary assessments, AI-based, disease management, mobile phone

## Abstract

**Background:**

Accurate measurement of food and nutrient intake is crucial for nutrition research, dietary surveillance, and disease management, but traditional methods such as 24-hour dietary recalls, food diaries, and food frequency questionnaires are often prone to recall error and social desirability bias, limiting their reliability. With the advancement of artificial intelligence (AI), there is potential to overcome these limitations through automated, objective, and scalable dietary assessment techniques. However, the effectiveness and challenges of AI applications in this domain remain inadequately explored.

**Objective:**

This study aimed to conduct a scoping review to synthesize existing literature on the efficacy, accuracy, and challenges of using AI tools in assessing food and nutrient intakes, offering insights into their current advantages and areas of improvement.

**Methods:**

This review followed the PRISMA-ScR (Preferred Reporting Items for Systematic Reviews and Meta-Analyses extension for Scoping Reviews) guidelines. A comprehensive literature search was conducted in 4 databases—PubMed, Web of Science, Cochrane Library, and EBSCO—covering publications from the databases’ inception to June 30, 2023. Studies were included if they used modern AI approaches to assess food and nutrient intakes in human subjects.

**Results:**

The 25 included studies, published between 2010 and 2023, involved sample sizes ranging from 10 to 38,415 participants. These studies used a variety of input data types, including food images (n=10), sound and jaw motion data from wearable devices (n=9), and text data (n=4), with 2 studies combining multiple input types. AI models applied included deep learning (eg, convolutional neural networks), machine learning (eg, support vector machines), and hybrid approaches. Applications were categorized into dietary intake assessment, food detection, nutrient estimation, and food intake prediction. Food detection accuracies ranged from 74% to 99.85%, and nutrient estimation errors varied between 10% and 15%. For instance, the RGB-D (Red, Green, Blue-Depth) fusion network achieved a mean absolute error of 15% in calorie estimation, and a sound-based classification model reached up to 94% accuracy in detecting food intake based on jaw motion and chewing patterns. In addition, AI-based systems provided real-time monitoring capabilities, improving the precision of dietary assessments and demonstrating the potential to reduce recall bias typically associated with traditional self-report methods.

**Conclusions:**

While AI demonstrated significant advantages in improving accuracy, reducing labor, and enabling real-time monitoring, challenges remain in adapting to diverse food types, ensuring algorithmic fairness, and addressing data privacy concerns. The findings suggest that AI has transformative potential for dietary assessment at both individual and population levels, supporting precision nutrition and chronic disease management. Future research should focus on enhancing the robustness of AI models across diverse dietary contexts and integrating biological sensors for a holistic dietary assessment approach.

## Introduction

Measuring food and nutrient intake is foundational in nutrition research, dietary surveillance, and clinical practice [1]. Traditional methods, such as 24-hour dietary recalls, food diaries, and food frequency questionnaires, have been the cornerstones of such endeavors [2]. However, these self-reported tools frequently encounter issues associated with recall error, where individuals inadvertently omit, underreport, or exaggerate certain food items or quantities [3]. Furthermore, social desirability bias further complicates matters, with respondents potentially altering their reports to reflect what they perceive as more socially acceptable or healthier dietary habits [4]. While clinical measures in controlled environments, such as laboratories, offer higher accuracy, they have drawbacks [5]. These objective measures often entail labor-intensive processes, significant costs, and potential intrusiveness for participants [6]. Such constraints render them less suitable for large-scale, population-level studies or individuals seeking to personally monitor their food and nutrient intake for disease management and other health-related objectives [6]. In light of these challenges, there is an escalating interest in leveraging artificial intelligence (AI) to enhance the accuracy and feasibility of dietary intake assessment [7].

AI, a branch of computer science focusing on developing algorithms that simulate human cognitive functions, has shown transformative potential across diverse sectors [8]. In health-related research, AI’s ability to process vast amounts of data at incredible speeds and its adeptness at pattern recognition has made substantial strides in medical imaging, predictive modeling of disease outbreaks, and personalized medicine [9,10]. In the context of dietary assessment, AI offers several distinct advantages. First, it can potentially mitigate the biases inherent in self-reported methods by using image recognition to identify and quantify food items with minimal input from the user [11]. Advanced machine learning algorithms can analyze photographs of meals and provide instant, objective assessments of portion sizes and nutrient content [11,12]. In addition to image-based methods, AI techniques also use sound, jaw motion from wearable devices, and text data for dietary assessment. These methods provide diverse approaches to capture dietary intake, enhancing the accuracy and comprehensiveness of assessments. Second, AI can offer continuous, real-time monitoring, bridging the temporal gap in methods like 24-hour recalls [13]. Finally, while laboratory-based clinical measures are costly and labor-intensive, once developed, AI-driven tools can be scaled up relatively inexpensively, making them more feasible for large population studies and individual dietary tracking [14]. Given these attributes, AI emerges as a promising candidate to revolutionize the landscape of food and nutrient intake measurement.

While numerous reviews have covered objective measures of dietary intake, our review specifically focuses on the application of AI technologies in this field. This scoping review provides a comprehensive synthesis of recent advancements, highlights the unique challenges faced by AI methodologies, and identifies critical gaps that future research should address. Our work adds to the existing literature by providing a detailed analysis of AI’s role in improving the accuracy and efficiency of dietary assessment.

To the best of our knowledge, a comprehensive scoping review that delves into the applications of AI for measuring food and nutrient intakes has not yet been conducted. This gap in the literature underlines the novelty and urgency of our investigation. The primary objective of this review is to explore and map out the current landscape of AI applications in dietary assessment, detailing methodologies, tools, and their associated findings.

This endeavor holds transformative potential for several reasons. First, by consolidating and synthesizing the vast yet dispersed body of knowledge, researchers, clinicians, and policy makers can gain a cohesive understanding of the current state-of-the-art and its implications for the future. Second, the review will spotlight any existing limitations or gaps in the current AI methodologies, paving the way for targeted advancements in technology and research design. Finally, given the paramount importance of accurate dietary assessment in myriad health outcomes and policy decisions, our findings can directly inform best practices, promote technology adoption in clinical and research settings, and guide future funding and priorities in technological and nutritional research sectors.

## Methods

### Overview

This scoping review followed the guidelines of the PRISMA-ScR (Preferred Reporting Items for Systematic Reviews and Meta-Analyses extension for Scoping Reviews; see Multimedia Appendix 1) [15].

### Study Selection Criteria

Predefined inclusion and exclusion criteria were established and applied to all identified studies during the screening process. Textbox 1 provides a detailed overview of the inclusion and exclusion criteria, outlining the study characteristics considered for eligibility in this review.

**Inclusion and exclusion criteria for study selection.**

Inclusion criteria:

- Study design: Experimental studies (eg, randomized controlled trials [RCTs], pre-post interventions) and observational studies (eg, cross-sectional, longitudinal).
- Analytic approach: Modern AI approaches, including machine learning (ML), deep learning (DL), and reinforcement learning (RL).
- Participants: Individuals of all ages.
- Data type: Input data, including food images, plate images, etc.
- Outcome: Measures on food and nutrient intakes.
- Article type: Original, empirical, peer-reviewed journal publications.
- Language: Articles written in English.
- Search time frame: From the inception of electronic bibliographic databases to June 30, 2023.

Exclusion criteria

- Study design: Studies that do not involve human subjects, observational or experimental design.
- Analytic approach: Studies using rule-based (“hard-coded”) approaches instead of example-based ML, DL, or RL.
- Participants: Non-human subjects.
- Data type: Studies not using dietary input data.
- Outcome: Studies without outcomes related to food and nutrient intakes.
- Article type: Letters, editorials, study or review protocols, case reports, or review articles.
- Language: Non–English-language articles.
- Search time frame: Studies published after June 30, 2023.

### Search Strategy

A comprehensive search was performed in 4 electronic bibliographic databases: PubMed, Web of Science, Cochrane Library, and EBSCO. The search strategy used a combination of controlled vocabulary (eg, MeSH terms in PubMed) and free-text keywords. The search terms were structured around two main concepts: (1) AI and (2) nutrition or dietary intake. The AI-related terms included: “artificial intelligence,” “machine learning,” “deep learning,” “neural networks,” “natural language processing,” “computer vision,” “algorithms,” “data mining,” “big data,” “predictive modeling,” and “automated pattern recognition.” The nutrition-related terms included: “nutrition,” “dietetics,” “nutritional sciences,” “diet,” “dietary behavior,” “beverage intake,” “food intake,” “nutrient intake,” and “healthy eating.” These keywords were combined using Boolean operators (AND, OR) to ensure a comprehensive search. The complete search strategy, including database-specific modifications and detailed search strings, is provided in Multimedia Appendix 2. After the initial search, 2 coauthors independently screened the titles and abstracts for the articles found through the keyword search, obtained potentially relevant articles, and reviewed their full texts. The inter-rater agreement between these two authors was evaluated using Cohen κ (κ=0.85). Disagreements were settled through conversation.

### Data Extraction and Synthesis

The following methodological and outcome variables were collected from each study using a standardized data extraction form: authors, year of publication, country or region, study objective, sample size, sample characteristics, AI models used, tasks and applications, type of input data, outcome measures, and perceived usefulness of AI technologies. No meta-analysis was feasible, given the substantial heterogeneity of the models, outcome measures, and applications. Therefore, we synthesized the study findings narratively and categorized them into distinct themes.

## Results

### Identification of Studies

**Figure 1:**
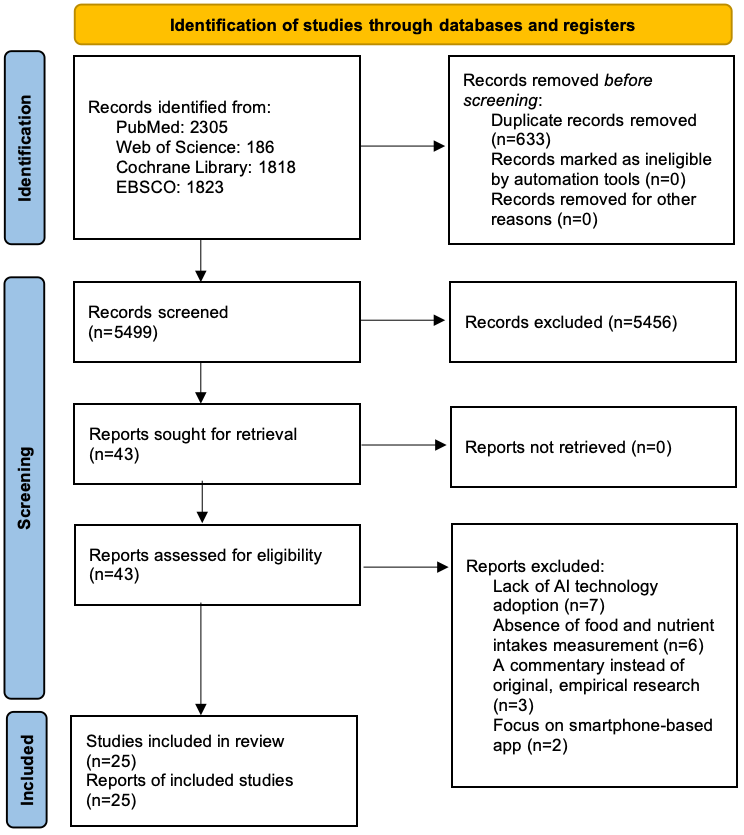
PRISMA (Preferred Reporting Items for Systematic reviews and Meta-Analyses) flow diagram illustrating the study selection process.

Figure 1 illustrates the PRISMA (Preferred Reporting Items for Systematic Reviews and Meta-Analyses) flow diagram, outlining the structured literature search and selection procedure. The initial database search identified 6132 articles. After removing duplicates, 5499 unique articles were retained for preliminary screening based on their titles and abstracts. From this collection, 5456 articles were evaluated as irrelevant and, consequently, excluded from the review. Applying the study selection criteria to the remaining 43 articles resulted in the further exclusion of 18 studies due to various reasons, including lack of AI technology adoption (n=7), absence of food and nutrient intake measurements (n=6), being a commentary rather than original empirical research (n=3), and a focus on smartphone-based apps (n=2). Ultimately, 25 studies met the relevance criteria and were included in the review [12,14,16-38].

### Study Characteristics

Table 1 reports the characteristics, type of input data, outcome measures, and main findings of the 25 studies incorporated in the review (more details in Multimedia Appendix 3). The studies spanned a range of publication years, with the earliest appearing in 2010 [16] and singular studies being published in 2013 [17], 2015 [18], and 2023 [38]. Publications in the following years were more frequent, with 2 studies each in 2016 [19,20], 2018 [21,22], and 2020 [14,27], 3 in 2021 [28-30], 5 in 2019 [12,23-26], and 7 in 2022 [31-37]. The geographical spread of the studies was diverse, with research conducted in several different countries: 14 in the United States [12,16,17,19-22,24-27,30,31,37], 4 in Switzerland [14,18,23,29], 2 in France [32,36], and 1 each in Canada [33], China [38], Denmark [34], Philippines [35], and Slovenia [28].

**Table 1:** Geographic location, sample size, sample characteristics, artificial intelligence models, type of input data, task, outcome measures, and main findings in the studies included in the review.

Author, Year	Country or Region	Sample size	Sample characteristics	AI^a^ models	Type of input data	Task	Outcome measures
Lopez-Meyer et al, 2010 [16]	United States	18	Healthy adults (BMI: 28.01, SD 6.35)	SVM^b^, RBFk^c^	Sound, strain signal	Classification	Food intake
Fontana et al, 2013 [17]	United States	12	Healthy adults (BMI: 24.39, SD 3.81)	RF^d^	Jaw motion signal, hand gesture signal, body acceleration	Classification	Food intake
Anthimopoulos et al, 2015 [18]	Switzerland	144	Images of dishes	CV^e^, SVM	Image	Regression	Carbohydrates counting
Farooq and Sazonov, 2016 [19]	United States	10	Healthy adults (BMI: 27.87, SD 5.51)	SVM, DT^f^	Jaw motion signal, body acceleration signal	Classification	Food intake
Hezarjaribi et al, 2016 [20]	United States	10	—^g^	SRM^h^, NLP^i^, SMM^j^	Audio signal	Regression	Calorie intake
Goldstein et al, 2018 [21]	United States	12	Adults with overweight/obesity (BMI: 33.60, SD 5.66)	RF, DT, Logit.Boot, BN^k^, Bagging, Random subspace	Text	Regression	Dietary lapses
Hezarjaribi et al, 2018 [22]	United States	30	—	NLP, LA^l^	Audio signal	Regression	Calorie intake
Lu et al, 2019 [23]	Switzerland	644	Meal images (pixel: 640×480)	MTNnet^m^, DTM^n^, RANSAC algorithm	Image	Regression	Nutrient intake
Fang et al, 2019 [12]	United States	4190	Food images (pixel: 224×224)	GAN^o^, CNN^p^	Image	Regression	Food energy
Jia et al, 2019 [24]	United States	38,415	Images (pixel: 640×480)	CNN	Image	Regression	Dietary assessment
Chin et al, 2019 [25]	United States	567	Food descriptions	LASSO^q^, Ridge, FFNN^r^, XGB^s^ models	Text	Regression	Amount of lactose
Farooq et al, 2019 [26]	United States	40	Healthy adults (BMI: 26.1, SD 5.2)	NNC^t^	Hand gesture, jaw motion, body acceleration	Classification	Food intake
Heremans et al, 2020 [27]	United States	126	Adults with dyspepsia	ANN^u^	Heart rate variability signal	Classification	Food intake
Lu et al, 2021 [14]	Switzerland	644	Meal images (pixel: 640×480)	MTCNet^v^, FSLBC^w^, 3D-SCA^x^	Image	Regression	Nutrient intake
Mezgec and Koroušić Seljak, 2021 [28]	Slovenia	520	Food images (pixel: 512×512)	DNN^y^	Image	Classification	Dietary assessment
Papathanail et al, 2021 [29]	Switzerland	866	Meal images (pixel: 640×480)	CNN, PSPNet^z^, DeepLabv3 network	Image	Regression	Energy, nutrient intake
Taylor et al, 2021 [30]	United States	34	Healthy adults (mean BMI: 24)	CNN, SMM	Text, voice data	Regression	Energy intake
Ghosh and Sazonov, 2022 [31]	United States	17	Adolescents and adults	Time-CNN, ResNet^aa^, FCN^ab^, IM^ac^, MLP^ad^	Accelerometer, optical sensor data	Classification	Food intake
Van Wymelbeke-Delannoy et al, 2022 [32]	France	22,544	Dishes images	DNN	Food image	Regression	Food item
Pfisterer et al, 2022 [33]	Canada	689	Plate images (pixel: 640×480)	Deep-CNN	Plate image	Regression	Food intake
Pedersen et al, 2022 [34]	Denmark	100	Adults with normal weight	RF	Psychophysiological responses	Regression	Food intake
Siy Van et al, 2022 [35]	Philippines	618	Children	RF, SVM, LDA^ae^, LR^af^	Text	Regression	Under-nutrition
Granal et al, 2022 [36]	France	375	Adults with chronic kidney disease	BN, BTANN^ag^	Text	Regression	Dietary potassium intake
Nguyen et al, 2022 [37]	United States	36	Adolescent	Pop-socket	Image, text	Regression	Dietary intake
Shao et al, 2023 [38]	China	5920	Food images	RGB-D^ah^ fusion network	Image	Regression	Energy, nutrient intake

^a^AI: artificial intelligence.

^b^SVM: support vector machine.

^c^RBFk: radial basis function kernels.

^d^RF: random forest.

^e^CV: computer vision.

^f^DT: decision tree.

^g^Not applicable.

^h^SRM: speech recognition model.

^i^NLP: natural language processing.

^j^SMM: string matching module.

^k^BN: Bayesian network.

^l^LA: levenshtein algorithm.

^m^MTNnet: multi-task neural network.

^n^DTM: delaunay triangulation method.

^o^GAN: generative adversarial networks.

^p^CNN: convolutional neural network.

^q^LASSO: least absolute shrinkage and selection operator.

^r^FFNN: feed forward neural network.

^s^XGB: eXtreme gradient boosting.

^t^NNC: neural network classifier.

^u^ANN: artificial neural network.

^v^MTCNet: multi-task contextual network.

^w^FSLBC: few-shot learning-based classifier.

^x^SCA: surface construction algorithm.

^y^DNN: deep neural network.

^z^PSPNet: pyramid scene parsing network.

^aa^ResNet: residual neural network.

^ab^FCN: fully convolutional neural network.

^ac^IM: inception network.

^ad^MLP: multilayer perceptron.

^ae^LDA: linear discriminant analysis.

^af^LR: logistic regression.

^ag^BTANN: bayesian tree augmented naive network.

^ah^RGB-D: Red, Green, Blue-Depth.

The studies varied in sample sizes, ranging from 10 to 38,415. Specifically, 10 studies had sample sizes between 10 and 99 [16,17,19-22,26,30,31,37], 3 had between 100 and 199 [18,27,34], and the remaining 12 had sample sizes exceeding 300. Among the 25 studies, while all involved human subjects, 10 studies focused on analyzing food images, dish images, or plate images to estimate dietary intake [12,14,18,23,24,28,29,32,33,38], 4 targeted healthy adults dealing with obesity or overweight [16,19,21,26], 3 focused on adults with normal weight [17,30,34], 3 engaged with children and adolescents [31,35,37], and 2 addressed adults with diseases [27,36]. Over the years, there have been notable advancements in AI-based dietary assessment. Early studies primarily focused on developing basic image recognition algorithms. More recent studies have integrated advanced machine learning models, such as deep learning and convolutional neural networks, which have significantly improved the accuracy of food recognition and nutrient estimation.

Among the 25 studies, 10 used image data, 9 used sound or jaw motion data from wearable devices, 4 used text data, and the remaining 2 combined multiple types of input data for dietary assessment. We classified the applications into 4 categories: dietary intake assessment, food detection, nutrient estimation, and food intake prediction.

### Applications in Dietary Intake Assessment

Our review identified several critical steps involved in the processing of dietary intake assessment systems, specifically for image-based methods. These steps include (1) identifying images with food, (2) identifying the foods, (3) separating the foods into separate parts, (4) estimating portion sizes served and remaining to estimate intake, and (5) estimating nutrient intake. Each of these steps involves distinct AI methodologies with varying degrees of accuracy and potential errors.

#### Identifying Images With Food

AI models, particularly convolutional neural networks (CNNs), are widely used for recognizing the presence of food in images. Studies, such as those by Fang et al (2019) [12] and Jia et al (2019) [24], have demonstrated high accuracy in detecting food presence using end-to-end image-based automatic food energy estimation techniques and real-world egocentric images, respectively.

#### Identifying the Foods

Once food is identified in an image, the next step is to classify and recognize different food items. Techniques such as support vector machines (SVMs) and deep learning models, including GANs (Generative Adversarial Networks) and advanced CNNs, are used for this purpose. For example, the GoCARB system developed by Anthimopoulos et al [18] used computer vision to estimate carbohydrate content by recognizing different food items from smartphone images.

#### Separating Foods Into Separate Parts

Segmenting individual food items within an image is crucial for accurate portion size estimation. Techniques such as image segmentation using deep neural networks (DNNs) have been effective in this regard. The study by Mezgec and Koroušić Seljak [28] showcased the use of DNNs for image-based dietary assessment with a classification accuracy of 86.72%.

#### Estimating Portion Sizes Served and Remaining

Estimating the portion sizes of served and remaining food requires precise volume and area measurements, which can be challenging due to varying presentation and occlusion of food items. AI models using RGB-D (Red, Green, Blue-Depth) imagery, as seen in the work by Shao et al [38], have shown promise in improving the precision of such estimations by using depth information to enhance the accuracy of food volume assessments.

#### Estimating Nutrient Intake

The final step involves estimating the nutrient intake based on the identified and quantified food items. This step often leverages databases such as the US Department of Agriculture (USDA) nutritional database to map food items to their nutrient profiles. The integration of AI for this purpose is exemplified by systems like the S2NI platform, which combines speech recognition and natural language processing to monitor dietary composition from spoken data, achieving high accuracy in nutrient computation.

Non–image-based dietary assessment methods, including those using sound, jaw motion from wearable devices, and text analysis, can also be categorized similarly. These methods contribute to various steps, particularly in identifying food intake and estimating nutrient content. For instance, the use of jaw motion signals analyzed by SVMs, as studied by Lopez-Meyer et al [16], provides high accuracy in detecting food intake.

### Applications in Food Detection

Food detection refers to the identification and recognition of food items using AI technologies. AI applications have become increasingly important in automating food detection, providing foundational advancements crucial for accurate nutrient estimation and food intake prediction. SVM and random forests are highlighted as prevalent machine learning models across the studies, aiming to achieve high food detection accuracy [16,17,19]. Random forest classification emphasizes the importance of time and frequency domain features in food intake detection with wearable sensor systems, focusing predominantly on jaw motion and accelerometer signals [17].

Another essential facet in this AI-infused dietary landscape is the integration of image-based assessments [28,33]. The development and validation of deep neural networks like NutriNet for food and beverage image recognition have showcased the ability of image-based approaches to identify multiple food or beverage items in a single image. Moreover, incorporating FCNs and deep residual networks (ResNet) magnifies the efficacy of segmenting food images, presenting a robust method in automated dietary assessments. Notably, Pfisterer et al [33] offered insights into the application of deep convolutional encoder-decoder food networks with depth-refinement (EDFN-D) in long-term care settings, providing an automated imaging system for quantifying food intake with high precision and objectivity, addressing the existing limitations in these settings [33].

A noticeable trend across the studies is the use of wearable and mobile devices, demonstrating the integration of technology with daily human activities for real-time and accurate data collection [19,24,32,37]. Wearable devices, such as the Automatic Ingestion Monitor (AIM) and other novel devices with sensors on the temporalis muscle and accelerometers, have shown potential in reducing the influence of motion artifacts and speech on food intake detection accuracy [19]. Furthermore, mobile AI technologies, such as FRANI (Food Recognition Assistance and Nudging Insights), illustrate their feasibility and reliability in resource-constrained settings, offering a comparable alternative to traditional methods like weighed records (WRs) [37].

DNN and CNN are central in recognizing and detecting food items from images, providing an automated approach to food detection and segmentation. The FoodIntech system, using a DNN-based approach, has demonstrated reliability in recognizing a variety of dishes and assessing food consumption [32]. Similarly, algorithms designed for egocentric images from wearable cameras have achieved substantial accuracy in food detection, addressing concerns related to data processing burdens and privacy [24].

Combining AI with RGB-D imagery is an evolving approach, showing promise in refining the precision of food nutrition estimation. The use of RGB-D fusion networks has revealed advancements in performing multimodal and multiscale feature fusion, offering a refined accuracy in nutrient analysis [38]. This approach successfully estimated calories and mass with a lower percentage mean absolute error and effectively visualized the estimation results of 4 nutrients [38].

Despite the advancements, there is a discernable disparity in the reported accuracy and reliability among the studies, with accuracy ranging from 74% to 99.85% [19,24]. This variance reflects the diverse methodologies, sensor modalities, ML algorithms, and the nature of features extracted for analysis. The ongoing refinements in methods and technologies showcase the evolving nature of AI applications in food detection, signaling a step forward in automating dietary assessment in varied environments and demographic settings.

### Applications in Nutrient Estimation

AI has been used to address the challenges associated with accurate nutrient intake assessment and dietary management for various medical conditions and patient demographics. The GoCARB system [18] exemplifies how AI can assist individuals with type 1 diabetes in carbohydrate counting, using computer vision to automate the estimation process using smartphones, hence aiding in optimal insulin dosage estimations. This application relies on the segmentation and recognition of food items, calculating the carbohydrate content based on food volumes and the USDA nutritional database, demonstrating a mean absolute percentage error in carbohydrate estimation of approximately 10%.

In addressing the nutrition assessment needs of hospitalized patients, an AI-based system has been developed [23,29] that uses RGB-D image pairs to estimate nutrient intake. These applications offer a means to counter malnutrition risks in hospital settings by delivering more accurate and automated nutrient intake assessments. The systems segment images into different food components, estimate the volume consumed, and calculate energy and macronutrient intake, showing a 15% estimation error [23] and improved agreement with expert estimations compared to standard clinical procedures [29].

Efforts have also been made to estimate food energy values using GAN architecture [12]. By mapping food images to their energy distributions, the technology has shown promise in improving the accuracy of dietary assessments, with an average error of 209 kcal per eating occasion in a real-world study setting.

In the context of 24-hour food recalls, machine learning models and database matching have been instrumental in estimating nutrients not directly outputted by specific dietary assessment tools [25]. For instance, lactose was relatively accurately estimated using models like XGB regressor and database matching methods.

Meanwhile, studies on the interplay between behavioral and physiologic variables in predicting food intake [34] have provided foundational insights. However, the predictive capability of combined or separate measures of food reward or biometric responses has not outperformed traditional models in clinical settings. The approach, however, lays the groundwork for further exploration of behavioral nutrition and personalized nutrition strategies.

Furthermore, the development of predictive tools leveraging AI for patients with chronic kidney disease has exhibited the potential to estimate dietary potassium intake, emphasizing the role of AI in clinical and therapeutic management [36]. This application has been noteworthy for its ability to classify potassium diet in 3 classes of potassium excretion with 74% accuracy, focusing more on clinical characteristics and renal pathology than on the potassium content of the ingested food.

Using mobile platforms that incorporate speech and natural language processing to convert spoken data to nutrient information offers a lens into the transformative potential of voice-based solutions [20,22,30]. These solutions, such as S2NI, Speech2Health, and the COCO Nutritionist app, achieve substantial accuracy in computing calorie intake, emphasizing the importance of real-time and pervasive monitoring. They demonstrate an integrated approach to capture dietary information more frequently, revealing the user preference toward voice-based interfaces over text-based and image-based nutrition monitoring due to their ease of use and accessibility.

### Applications in Food Intake Prediction

Food intake prediction involves estimating the amount and type of food consumed based on detected items. Advancements in AI are significantly shaping the landscape of food intake prediction by offering various innovative solutions and techniques. For instance, ML techniques in predicting dietary lapses during weight loss interventions have demonstrated the potential to augment adherence to dietary guidelines and offer real-time interventions, providing a comprehensive perspective on combining individual and group-level data to enrich predictions [21].

The adaptability and efficiency of ML are further highlighted in the studies focusing on detecting food intake using various sensor technologies and algorithms. Developing and validating sensor-based food intake detection methods, such as AIM, have illustrated high accuracy and reliability, presenting a promising future for food intake monitoring in unconstrained environments [26,31]. SVMs have been effectively used in monitoring ingestive behavior, yielding up to 94% accuracy in detecting food intake by analyzing chews and swallows [16].

In particular, the utility of DL algorithms, like ResNet and Fully Convolutional Neural Network (FCN), is revealed to be paramount in differentiating food intake from other activities using sensor signals. The competitive performance of these algorithms indicates the significance of selecting appropriate methods for precise classifications in real-world scenarios, establishing their importance in the evolving field of dietary monitoring and health interventions [31].

The exploration of DNN in automatic food intake detection through dynamic analysis of heart rate variability has opened avenues for addressing meal-related disorders. The notable accuracy of DNN, especially in neuromodulation treatments for conditions like obesity and diabetes, establishes the potential of ML in contributing to varied health care settings [27].

Furthermore, the studies using ML algorithms like the random forest have provided a robust method for identifying and comparing nutritional risk, offering valuable insights into developing targeted nutritional interventions and effectively addressing undernutrition. Such approaches are crucial in considering local dietary culture and delivering more nuanced and culturally competent health care solutions [35].

Another essential facet in this AI-infused dietary landscape is the integration of image-based assessments [28,33]. The development and validation of deep neural networks like NutriNet for food and beverage image recognition have showcased the ability of image-based approaches to identify multiple food or beverage items in a single image. Furthermore, incorporating FCNs and deep residual networks (ResNet) magnifies the efficacy of segmenting food images, presenting a robust method in automated dietary assessments. Notably, Pfisterer et al [33] offered insights into the application of deep convolutional EDFN-D in long-term care settings, providing an automated imaging system for quantifying food intake with high precision and objectivity, addressing the existing limitations in these settings [33].

## Discussion

### Principal Findings

The increasing intersection of AI with dietary assessment has emerged as a transformative trend, as evidenced by our scoping review. Our literature search revealed 25 pertinent studies published between 2010 and 2023. These studies spanned several nations, diverse demographics, and a spectrum of methodologies. At its core, AI has primarily been used in 3 domains: food detection, nutrient estimation, and food intake prediction. Machine learning models like SVMs and random forests and deep learning models like CNNs have proved instrumental in enhancing the accuracy of food detection and nutrient estimation, often integrated with wearable devices and mobile platforms. Another observation was the use of AI in designing user-friendly interfaces, such as voice-based inputs, to improve adherence to dietary tracking. User experience with AI-based dietary assessment tools varies, but studies indicate generally positive feedback regarding ease of use and convenience. Users appreciate the real-time feedback and reduced burden of manual input. However, there are concerns about accuracy and privacy. Enhanced user training and transparent data privacy policies could improve user trust and interaction with these tools. The collective findings underscore the potential of AI to revolutionize dietary assessment, providing robust accuracy and user-centric solutions. This amalgamation of technology and nutrition research addresses the inherent limitations of traditional methods and charts a path for more personalized, accurate, and real-time dietary assessments in varied settings.

As illustrated by the reviewed studies, integrating AI into food and nutrient intake assessments showcases a marked advancement over traditional methodologies commonly used in nutritional science [14,37]. Historically, methods such as 24-hour recalls, food frequency questionnaires, and dietary records have been the mainstream of dietary assessments [2]. While these methods have provided invaluable insights, they have inherent limitations like recall bias, inaccuracies stemming from self-reporting, and the logistical challenges of frequent, detailed data recording [3]. The reviewed studies, however, highlighted the significant potential of AI to alleviate some of these concerns. For instance, AI-backed systems such as FRANI have been shown to offer a reliable alternative to weighed records, which, although thorough, can be burdensome for participants [37]. Similarly, tools like the GoCARB system automate carbohydrate counting, which, if done manually, demands meticulous attention and can be prone to errors, especially for individuals with conditions like diabetes [18].

Furthermore, the versatility of AI applications across various nutritional assessments is evident from the reviewed literature. For instance, SVMs and random forests, when deployed in monitoring ingestive behaviors, have demonstrated high accuracy in detecting food intake by analyzing nuances such as chews and swallows [16]. This level of precision is difficult to attain through manual observation or self-reports. Applying DNNs to recognize food items from images underscores another leap, automating a process that traditionally demands human expertise. Furthermore, the intersection of AI with RGB-D imagery suggests an improved accuracy in nutrient analysis, an area where traditional methods may not always yield precise results [38]. However, it is crucial to note the variability in reported accuracy among studies, which underscores the importance of refining methodologies and recognizing the evolving nature of AI applications. Despite this, the current trajectory indicates that AI is poised to bring a paradigm shift in automating dietary assessment, melding accuracy with efficiency [36,37]. Wearable technology that detects food intake based on chews and swallows offers significant benefits in real-time dietary monitoring, particularly in clinical and research settings. These devices can be integrated with mobile applications and other wearable sensors to provide comprehensive dietary assessments. While continuous camera use may not be practical for all users, advancements in discreet wearable sensors and intermittent image capture can enhance user compliance and accuracy.

While AI’s promise in food and nutrient intake measurement is evident, its application comes with intrinsic challenges and limitations. The reviewed studies, as well as the broader literature, highlight some consistent concerns. First, the AI models heavily depend on the quality and breadth of training data [39]. A model trained on a limited dataset may not recognize diverse food items, particularly those from various global cuisines or those prepared using unique methods [40]. This can lead to inaccuracies in nutrient estimation. Common biases include algorithmic biases resulting from non-diverse training datasets that fail to represent global food diversity. In addition, limitations in image-based recognition systems often stem from varying image quality and presentation, which can affect the accuracy of food and nutrient estimations. The variability in food presentation, portion sizes, and the physical environment in which the food is captured (eg, lighting conditions) can pose challenges for image-based recognition systems [41,42]. Furthermore, while tools like FRANI and GoCARB show promise, they also underscore the current limitations in recognizing mixed dishes or deciphering layered foods with multiple ingredients [18,37]. It is also worth noting that AI systems while reducing human biases, introduce computational biases that may arise from algorithmic designs or training datasets [43,44]. These challenges highlight the need for more comprehensive datasets and improved image processing techniques to enhance AI model reliability. Finally, a potential digital divide exists, where populations without access to advanced technology or those not adept at using it might be excluded from AI-based dietary assessments, thereby limiting its universal applicability [45,46].

Many AI-based dietary assessment tools rely on dietitians to validate and estimate dietary intake from images due to the complexities involved in accurate food identification and portion size estimation. With the constant addition of new food items, maintaining up-to-date nutrient databases is challenging. Some studies have focused narrowly on estimating energy intake or working with a limited set of foods under controlled conditions, which limits the generalizability of their findings. Future research should focus on developing scalable AI models that can handle a broader range of foods and integrate real-time updates to nutrient databases. In addition, enhancing the collaboration between AI technologies and dietitians can help improve the accuracy and applicability of these tools.

Current objective methods face significant limitations, including inaccuracies in nutrient composition tables, the complexity of multi-ingredient dishes, and variability in nutrient composition of commercially available foods. In addition, these methods do not account for individual metabolic differences in nutrient processing. Integrating biological sensors with AI technologies could offer a more definitive approach by providing real-time data on circulating nutrients and individual metabolic responses, thereby improving the accuracy of dietary assessments.

The sequential nature of AI-based dietary assessment introduces cumulative errors, where inaccuracies at each stage—from food detection to nutrient estimation—can compound, leading to significant overall errors. Biological sensors that measure circulating nutrients in real-time offer a promising solution to overcome these limitations, as they provide direct data on nutrient absorption and metabolism, reducing reliance on intermediate estimations and improving overall accuracy.

Our search strategy, while comprehensive, may not have captured all studies involving AI and dietary assessment. Despite significant advancements, several gaps remain in the application of AI for dietary assessment. Future research should focus on enhancing the diversity of training datasets to reduce algorithmic biases and improve the accuracy of AI models in recognizing a wide variety of food items. In addition, integrating real-time metabolic data with dietary assessments could offer more comprehensive insights into individual nutritional statuses. Among the AI tools evaluated, image-based recognition systems like the GoCARB system are highly effective for carbohydrate counting in diabetes management, while wearable devices monitoring jaw motion offer promising real-time intake data, particularly useful in clinical settings.

Ethical considerations in AI-based dietary assessment are paramount. Data privacy concerns arise from the extensive personal data required for accurate assessments, necessitating robust security measures and transparent consent processes. Algorithmic biases can lead to inaccuracies and unfair outcomes, highlighting the need for diverse training datasets. In addition, the digital divide poses a significant challenge, as populations without access to advanced technologies may be excluded from the benefits of AI. Addressing these issues requires comprehensive strategies, including inclusive technology design and stringent ethical standards in data handling and algorithm development.

As AI continues to evolve, there is vast potential for revolutionary enhancements in dietary and nutrient intake measurement. Based on current trajectories in nutrition science and AI advancements, we might anticipate a future where AI systems can recognize food items with high precision and factor in variables like cooking methods, regional variations, and the bioavailability of nutrients. These AI systems could be trained on increasingly diverse datasets, capturing the nuances of global diets and potentially integrating real-time metabolic and physiological data from wearable devices to provide a more comprehensive view of an individual’s nutrient absorption [47,48]. AI could facilitate large-scale dietary assessment studies on a population level, helping researchers discern dietary patterns, nutrient deficiencies, and even epidemiological correlations faster and more accurately [49,50]. With the rise of precision nutrition, AI might enable personalized dietary recommendations, considering an individual's genetic, metabolic, and health profile [51]. This tailored approach could radically improve disease management, particularly for conditions like diabetes or cardiovascular diseases, where dietary interventions play a pivotal role [52].

### Conclusion

In conclusion, the scoping review highlighted the burgeoning role of AI in advancing the measurement of food and nutrient intakes, with notable advancements in accuracy and efficiency compared to traditional methods. However, while the potential of AI in this domain is substantial, it is imperative to acknowledge its current limitations and areas requiring refinement. As the nexus between nutrition science and technology continues to strengthen, future research must focus on refining AI methodologies, ensuring their applicability across diverse populations, and integrating them into broader nutritional and health studies. This interdisciplinary collaboration promises a future where dietary assessments are accurate and instrumental in shaping individual and public health outcomes.

## Appendix

Multimedia Appendix 1 PRISMA (Preferred Reporting Items for Systematic Reviews and Meta-Analyses) checklist (Note that our paper is a scoping review rather than a systematic review, so some criteria in the checklist may not apply and are thus omitted).

Multimedia Appendix 2 Database search algorithms.

Multimedia Appendix 3 Main findings in the studies included in the review.

## Abbreviations

AI: artificial intelligence
AIM: Automatic Ingestion Monitor
CNN: convolutional neural network
DNN: deep neural network
EDFN-D: encoder-decoder food networks with depth-refinement
FCN: Fully Convolutional Neural Network
FRANI: Food Recognition Assistance and Nudging Insights
GAN: Generative Adversarial Network
PRISMA: Preferred Reporting Items for Systematic Reviews and Meta-Analyses
PRISMA-ScR: Preferred Reporting Items for Systematic Reviews and Meta-Analyses extension for Scoping Reviews
ResNet: residual networks
RGB-D: Red, Green, Blue-Depth
SVM: support vector machine
USDA: US Department of Agriculture
WR: weighed record

## Data Availability

Data sharing is not applicable to this article as no datasets were generated or analyzed during this study.
