# Supplementary material for: Artificial Intelligence Applications to Measure Food and Nutrient Intakes: Scoping Review
Source: J Med Internet Res. 2024 Nov 28;26:e54557. doi: 10.2196/54557 (PMC11638690; doi:10.2196/54557)
Supplement: Multimedia Appendix 2 [file jmir_v26i1e54557_app2.docx]

**Database Search Algorithms**

**1. PubMed:** (Used the following Filters: Humans, English, MEDLINE) (("Artificial Intelligence"[MeSH Terms] OR "Machine Learning"[MeSH Terms] OR "Deep Learning"[MeSH Terms] OR "neural networks, computer"[MeSH Terms] OR "Natural Language Processing"[MeSH Terms] OR "algorithms"[MeSH Terms] OR "data mining"[MeSH Terms] OR "big data"[MeSH Terms] OR "pattern recognition, automated"[MeSH Terms] OR "Deep Learning"[MeSH Terms] OR "Machine Learning"[MeSH Terms] OR "Natural Language Processing"[MeSH Terms] OR ("Artificial Intelligence"[Title/Abstract] OR "AI"[Title/Abstract] OR "Machine Learning"[Title/Abstract] OR "Deep Learning"[Title/Abstract] OR "neural networks"[Title/Abstract] OR "Natural Language Processing"[Title/Abstract] OR "computer vision"[Title/Abstract])) AND ("Dietetics"[MeSH Terms] OR "Nutritional Sciences"[MeSH Terms] OR "Diet"[MeSH Terms] OR "beverages"[MeSH Terms] OR "dietary behavior"[Title/Abstract] OR "beverage intake"[Title/Abstract] OR "beverage consumption"[Title/Abstract] OR "food intake"[Title/Abstract] OR "food consumption"[Title/Abstract] OR "nutrient intake"[Title/Abstract] OR "healthy eating"[Title/Abstract] OR "diet quality"[Title/Abstract])

**2. Web of Science:** (Refined by the following: Document Types: Articles; Languages: English) ((TI=("Artificial Intelligence" OR "Machine Learning" OR "Deep Learning" OR "Neural Networks, Computer" OR "Natural Language Processing" OR "Computer Vision" OR "Algorithms" OR "Data mining" OR "Big data" OR "Predictive modeling" OR "Pattern recognition, automated" OR "Artificial neural networks" OR "AI") AND TI=("Nutrition" OR "Dietetics" OR "Nutritional Sciences" OR "Diet" OR "Dietary behavior" OR "Beverages" OR "Food intake" OR "Nutrient intake" OR "Healthy eating" OR "Beverage intake" OR "Beverage consumption" OR "Food consumption" OR "Diet quality"))

**3. Cochrane Library:** ("Artificial Intelligence" OR "Machine Learning" OR "Deep Learning" OR "Neural Networks, Computer" OR "Natural Language Processing" OR "Computer Vision" OR "Algorithms" OR "Data mining" OR "Big data" OR "Predictive modeling" OR "Pattern recognition, automated" OR "Artificial neural networks" OR "AI") AND ("Nutrition" OR "Dietetics" OR "Nutritional Sciences" OR "Diet" OR "Dietary behavior" OR "Beverages" OR "Food intake" OR "Nutrient intake" OR "Healthy eating" OR "Beverage intake" OR "Beverage consumption" OR "Food consumption" OR "Diet quality")

**4. EBSCO:** ("Artificial Intelligence" OR "Machine Learning" OR "Deep Learning" OR "Neural Networks, Computer" OR "Natural Language Processing" OR "Computer Vision" OR "Algorithms" OR "Data mining" OR "Big data" OR "Predictive modeling" OR "Pattern recognition, automated" OR "Artificial neural networks" OR "AI") AND ("Nutrition" OR "Dietetics" OR "Nutritional Sciences" OR "Diet" OR "Dietary behavior" OR "Beverages" OR "Food intake" OR "Nutrient intake" OR "Healthy eating" OR "Beverage intake" OR "Beverage consumption" OR "Food consumption" OR "Diet quality")
