# Supplementary material for: Artificial Intelligence Applications to Measure Food and Nutrient Intakes: Scoping Review
Source: J Med Internet Res. 2024 Nov 28;26:e54557. doi: 10.2196/54557 (PMC11638690; doi:10.2196/54557)
Supplement: Multimedia Appendix 3 [file jmir_v26i1e54557_app3.docx]

Multimedia Appendix 3. Main findings in the studies included in the review.

| Author, Year | The Usefulness of AI Technologies |
| --- | --- |
| Lopez-Meyer, 2010 [1] | The highest accuracy of detecting food intake (94%) was achieved in a configuration where chews and swallows were used as predictors. Using only swallowing as a predictor resulted in 80% accuracy. |
| Fontana, 2013 [2] | Frequency domain features from the jaw motion signal and time domain features from the accelerometer signal were the most relevant features for food intake detection. |
| Anthimopoulos, 2015 [3] | The mean absolute percentage error in carbohydrates estimation was 10 ± 12%. |
| Farooq, 2016 [4] | This approach resulted in an average F1-score of 99.85% and an area under  the curve of 0.99 for multiclass classification. |
| Hezarjaribi, 2016 [5] | A comprehensive nutrition monitoring system achieves 80.6% accuracy in computing calorie intake. |
| Goldstein, 2018 [6] | The accuracy of predicting dietary lapses was 0.72. |
| Hezarjaribi, 2018 [7] | Accuracy was 92.2% in computing calorie intake. |
| Lu, 2019 [8] | The estimation error of nutrient intake for each food type was 15%. |
| Fang, 2019 [9] | The average error in the estimated energy was 209 kcal per eating occasion. |
| Jia, 2019 [10] | The AI technology can automatically detect foods from low-quality, wearable camera-acquired real-world egocentric images with reasonable accuracy. |
| Chin, 2019 [11] | Among machine learning models, the XGB-Regressor model performed best on held-out test data (R2 = 0.33). For the database matching method, nutrient + text matching yielded the best lactose estimates (R2 = 0.76). |
| Farooq, 2019 [12] | The validity of sensor-based food intake detection was 0.78±0.12, and there were no statistically significant differences among the average eating duration estimated from raters' annotations and sensor-based predictions (p-value = 0.19). |
| Heremans, 2020 [13] | The classification accuracy was 0.95-0.995 by ANN. |
| Lu, 2021 [14] | Estimated nutrient intake is highly correlated (>0.91) to the ground truth and shows small mean relative errors (<20%). |
| Mezgec, 2021[15] | The developed approach had a classification accuracy of 86.72% on the recognition dataset. |
| Papathanail, 2021[16] | For the energy calculation, the carbohydrate, protein, and fatty acids intake, the system's estimations had a mean error of only 41 kcal (11.64%), 4.6 g (13.23%), 1.4 g (10.47%), 1.9 g (11.70%) and 1.2 g (14.84%) per meal, respectively. |
| Taylor, 2021 [17] | There was no significant difference in energy intake between values obtained by the COCO app and 24-hour recall for days. |
| Ghosh, 2022 [18] | ResNet outperforms Time-CNN, FCN, ResNet, and Inception networks for this problem. |
| Wymelbeke-Delannoy, 2022 [19] | The reliability of the AI results appeared to be excellent for 39% of the dishes and good for 19%. |
| Pfisterer, 2022 [20] | A novel deep convolutional encoder-decoder food network with depth-refinement performed comparably to depth-refined graph cut on IOU (0.879 vs. 0.887), with intake errors well below typical 50% (mean percent intake error: −4.2%). |
| Pedersen, 2022 [21] | Combined or separate measures of food reward or biometric responses did not predict energy intake better than the naïve model. |
| Van, 2022 [22] | Random forest was the most accurate ML algorithm (78.55%), able to predict under-nutrition based on household expenditures, child and household age, food insecurity, and dietary diversity. |
| Granal, 2022 [23] | The accuracy of the prediction tool to classify potassium diet in the three classes of potassium excretion was 74%. |
| Nguyen, 2022 [24] | AI-assisted dietary assessment and 24HRs accurately estimated adolescent females' nutrient intake compared with weighted records. |
| Shao, 2023 [25] | The percentage mean absolute error of calories and mass reached 15.0% and 10.8% via the RGB-D fusion network, improved by 3.8% and 8.1%, respectively. |

**References**

1. Lopez-Meyer P, Schuckers S, Makeyev O, Sazonov E. Detection of periods of food intake using Support Vector Machines. Annu Int Conf IEEE Eng Med Biol Soc 2010; 2010:1004-1007.
2. Fontana JM, Farooq M, Sazonov E. Estimation of feature importance for food intake detection based on Random Forests classification. Annu Int Conf IEEE Eng Med Biol Soc 2013; 2013:6756-6759.
3. Anthimopoulos M, Dehais J, Shevchik S, Ransford BH, Duke D, Diem P, Mougiakakou S. Computer vision-based carbohydrate estimation for type 1 patients with diabetes using smartphones. J Diabetes Sci Technol 2015; 9(3):507-515.
4. Farooq M, Sazonov E. A novel wearable device for food intake and physical activity recognition. Sensors (Basel) 2016; 16(7):1067.
5. Hezarjaribi N, Reynolds CA, Miller DT, Chaytor N, Ghasemzadeh H. S2NI: a mobile platform for nutrition monitoring from spoken data. Annu Int Conf IEEE Eng Med Biol Soc 2016; 2016:1991-1994.
6. Goldstein SP, Zhang F, Thomas JG, Butryn ML, Herbert JD, Forman EM. Application of machine learning to predict dietary lapses during weight loss. J Diabetes Sci Technol 2018; 12(5):1045-1052.
7. Hezarjaribi N, Mazrouee S, Ghasemzadeh H. Speech2Health: a mobile framework for monitoring dietary composition from spoken data. IEEE J Biomed Health Inform 2018; 22(1):252-264.
8. Lu Y, Stathopoulou T, Vasiloglou MF, Christodoulidis S, Blum B, Walser T, Meier V, Stanga Z, Mougiakakou SG. An artificial intelligence-based system for nutrient intake assessment of hospitalised patients. Annu Int Conf IEEE Eng Med Biol Soc 2019; 2019:5696-5699.
9. Fang S, Shao Z, Kerr DA, Boushey CJ, Zhu F. An end-to-end image-based automatic food energy estimation technique based on learned energy distribution images: protocol and methodology. Nutrients 2019; 11(4):877.
10. Jia W, Li Y, Qu R, Baranowski T, Burke LE, Zhang H, Bai Y, Mancino JM, Xu G, Mao ZH, Sun M. Automatic food detection in egocentric images using artificial intelligence technology. Public Health Nutr 2019; 22(7):1168-1179.
11. Chin EL, Simmons G, Bouzid YY, Kan A, Burnett DJ, Tagkopoulos I, Lemay DG. Nutrient estimation from 24-hour food recalls using machine learning and database mapping: a case study with lactose. Nutrients 2019; 11(12):3045.
12. Farooq M, Doulah A, Parton J, McCrory MA, Higgins JA, Sazonov E. Validation of sensor-based food intake detection by multicamera video observation in an unconstrained environment. Nutrients 2019; 11(3):609.
13. Heremans ERM, Chen AS, Wang X, Cheng J, Xu F, Martinez AE, Lazaridis G, Van Huffel S, Chen JDZ. Artificial neural network-based automatic detection of food intake for neuromodulation in treating obesity and diabetes. Obes Surg 2020; 30(7):2547-2557.
14. Lu Y, Stathopoulou T, Vasiloglou MF, Christodoulidis S, Stanga Z, Mougiakakou S. An artificial intelligence-based system to assess nutrient intake for hospitalised patients. IEEE Trans. Multimedia 2021; 23:1136-1147.
15. Mezgec S, Koroušić Seljak B. Deep neural networks for image-based dietary assessment. J Vis Exp 2021; (169):e61906.
16. Papathanail I, Brühlmann J, Vasiloglou MF, Stathopoulou T, Exadaktylos AK, Stanga Z, Münzer T, Mougiakakou S. Evaluation of a novel artificial intelligence system to monitor and assess energy and macronutrient intake in hospitalised older patients. Nutrients 2021; 13(12):4539.
17. Taylor S, Korpusik M, Das S, Gilhooly C, Simpson R, Glass J, Roberts S. Use of natural spoken language with automated mapping of self-reported food intake to food composition data for low-burden real-time dietary assessment: method comparison study. J Med Internet Res 2021; 23(12):e26988.
18. Ghosh T, Sazonov E. A comparative study of deep learning algorithms for detecting food intake. Annu Int Conf IEEE Eng Med Biol Soc 2022; 2022:2993-2996.
19. Van Wymelbeke-Delannoy V, Juhel C, Bole H, Sow AK, Guyot C, Belbaghdadi F, Brousse O, Paindavoine M. A cross-sectional reproducibility study of a standard camera sensor using artificial intelligence to assess food items: the foodIntech project. Nutrients 2022; 14(1):221.
20. Pfisterer KJ, Amelard R, Chung AG, Syrnyk B, MacLean A, Keller HH, Wong A. Automated food intake tracking requires depth-refined semantic segmentation to rectify visual-volume discordance in long-term care homes. Sci Rep 2022; 12(1):83.
21. Pedersen H, Diaz LJ, Clemmensen KKB, Jensen MM, Jørgensen ME, Finlayson G, Quist JS, Vistisen D, Færch K. Predicting food intake from food reward and biometric responses to food cues in adults with normal weight using machine learning. J Nutr 2022; 152(6):1574-1581.
22. Siy Van VT, Antonio VA, Siguin CP, Gordoncillo NP, Sescon JT, Go CC, Miro EP. Predicting undernutrition among elementary schoolchildren in the Philippines using machine learning algorithms. Nutrition 2022; 96:111571.
23. Granal M, Slimani L, Florens N, Sens F, Pelletier C, Pszczolinski R, Casiez C, Kalbacher E, Jolivot A, Dubourg L, Lemoine S, Pasian C, Ducher M, Fauvel JP. Prediction tool to estimate potassium diet in chronic kidney disease patients developed using a machine learning tool: the univerSel study. Nutrients 2022; 14(12):2419.
24. Nguyen PH, Tran LM, Hoang NT, Trương DTT, Tran THT, Huynh PN, Koch B, McCloskey P, Gangupantulu R, Folson G, Bannerman B, Arrieta A, Braga BC, Arsenault J, Kehs A, Doyle F, Hughes D, Gelli A. Relative validity of a mobile AI-technology-assisted dietary assessment in adolescent females in Vietnam. Am J Clin Nutr 2022; 116(4):992-1001.
25. Shao W, Min W, Hou S, Luo M, Li T, Zheng Y, Jiang S. Vision-based food nutrition estimation via RGB-D fusion network. Food Chem 2023; 424:136309.
